# Supplementary material for: Simultaneous Quantitative Analysis of Six Isothiazolinones in Water-Based Adhesive Used for Food Contact Materials by High-Performance Liquid Chromatography–Tandem Mass Spectrometry (HPLC–MS/MS)
Source: Molecules. 2019 Oct 29;24(21):3894. doi: 10.3390/molecules24213894 (PMC6865086; doi:10.3390/molecules24213894)

The supplementary materials

Figure s1 Gradient elution program of mobile phase in qualitative analysis of standard solution of MI,CMI,BIT,MBIT,OIT and COIT

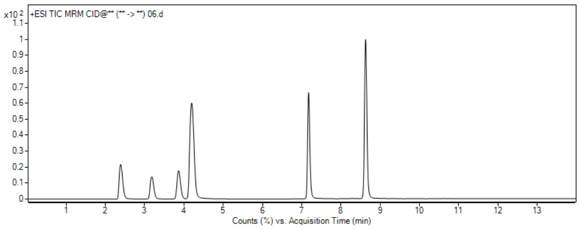

Time(min)	B:Methanol (%)	Flow(mL/min)
0	50	0.3
1	50	0.3
1.5	90	0.3
6	90	0.3
6.1	50	0.3
14	50	0.3

Time(min)	B:Methanol (%)	Flow(mL/min)
0	50	0.3
3	50	0.3
6	90	0.3
12	90	0.3
12.1	50	0.3
20	50	0.3

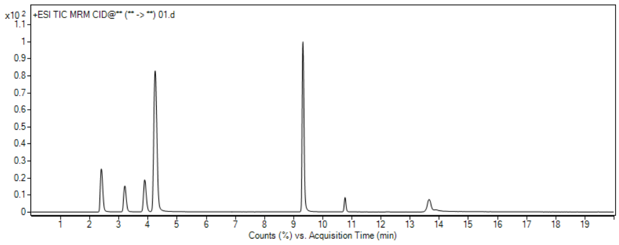

Supplement: Supplementary file 1 [file molecules-24-03894-s001.pdf]
